# Tsukushi and *TSKU* genotype in obesity and related metabolic disorders

**DOI:** 10.1007/s40618-021-01572-x

**Published:** 2021-04-15

**Authors:** Y. Li, L. Jin, J. Yan, Y. Huang, H. Zhang, R. Zhang, C. Hu

**Affiliations:** 1 Shanghai Key Laboratory of Diabetes Mellitus, Shanghai Clinical Centre for Diabetes, Shanghai Diabetes Institute, Shanghai Jiao Tong University Affiliated Sixth People’s Hospital, 600 Yishan Road, Shanghai, 200233 China; 2 Institute for Metabolic Disease, Fengxian Central Hospital Affiliated to The Third School of Clinical Medicine, Southern Medical University, Shanghai, China

**Keywords:** Tsukushi, *TSKU*, Single-nucleotide polymorphism, Obesity, Visceral fat area

## Abstract

**Purpose:**

Whether Tsukushi (TSK) can protect against high-fat diet (HFD)-induced obesity and improve glucose metabolism remains controversial. Serum levels of TSK in the population have not been reported until now. We assessed the association among TSK level, *TSKU* genotype, and metabolic traits in humans.

**Methods:**

Associations between serum TSK levels and metabolic traits were assessed in 144 Han Chinese individuals. Loci in the *TSKU* gene region were further genotyped in 11,022 individuals. The association between the loci and serum TSK level was evaluated using the additive genetic model. The association between the loci and their metabolic traits in humans were also verified.

**Results:**

Lower TSK levels were observed in obese subjects than in control subjects (median and interquartile range 17.78:12.07–23.28 vs*.* 23.81:12.54–34.56, *P* < 0.05). However, in obese subjects, TSK was positively associated with BMI (*β* ± SE: 0.63 ± 0.31, *P* = 0.049), visceral fat area (*β* ± SE: 12.15 ± 5.94, *P* = 0.011), and deterioration of glucose metabolism. We found that rs11236956 was associated with TSK level in obese subjects (*β* 95% CI 0.17, 0.07–0.26; *P* = 0.0007). There was also a significant association between rs11236956 and metabolic traits in our population.

**Conclusions:**

Our findings showed that serum TSK levels were associated with metabolic disorders in obese subjects. We also identified rs11236956 to be associated with serum TSK levels in obese subjects and with metabolic disorders in the total population.

**Supplementary Information:**

The online version contains supplementary material available at 10.1007/s40618-021-01572-x.

## Introduction

The liver is the principal organ in the regulation of systemic metabolism and energy homeostasis. It directly participates in the processes of uptake, synthesis, oxidation and secretion of lipid and glucose [1]. It is also recognized that the liver can secrete hepatokines to regulate other extrahepatic metabolic tissues, via autocrine, paracrine, and endocrine signaling [2]. The global prevalence of obesity has risen constantly and rapidly in recent years [3] and has triggered an increase in related metabolic diseases, such as non-alcoholic fatty liver disease (NAFLD), type 2 diabetes, dyslipidemia, hypertension, and cardiovascular disease. In the case of an energy metabolism disorder, the synthesis and secretion of hepatokines by the liver are consequently altered to exert a compensatory effect [2, 4, 5]. One hepatokine, FGF21, has been well documented as able to increase fatty acid *β*-oxidation, alleviate hepatocyte steatosis, decrease adipose tissue inflammation, and improve insulin sensitivity [6]. The further identification and functional characterization of hepatokines may provide significant insights into the pathogenesis of metabolic diseases and offer a potential therapeutic target.

Tsukushi (TSK) is a small, secreted protein of the leucine-rich proteoglycan family, encoded by the *TSKU* gene. It is an organizer inducer that is involved in the development of multiple tissues [7]. Using the methods of liver secretome analysis, and a comprehensive analytical platform, two groups have newly identified TSK as a hepatokine [8, 9]. It has been confirmed in multiple mouse models that the hepatic expression and elevated circulating levels of *Tsku,* induced by obesity, are correlated with NAFLD [9]. However, contradictory results have been obtained with regard to body weight, glucose metabolism, and thermogenesis. Wang et al. found that Tsk knockout mice exhibited marked resistance to obesity and metabolic disorders induced by a high-fat diet (HFD). Blood glucose levels were significantly lower in knockout mice than in the wild-type. Meanwhile, TSK deficiency promoted adipose thermogenesis and energy expenditure [8]. Conversely, another study found that TSK knockout mice were not protected against the development of obesity and did not show improvement in glucose tolerance [9, 10]. Nevertheless, TSK impacted lipid homeostasis by reducing circulating HDL cholesterol, lowering cholesterol efflux capacity, and decreasing cholesterol-to-bile acid conversion in the liver. However, modulating TSK expression had little effect on NAFLD development and progression [9, 10]. Paradoxical results have left the role of TSK in obesity and NAFLD uncertain.

To date, few studies have reported on TSK expression in the human liver [11]. One study has shown that liver TSK expression is positively correlated with human liver steatosis [9], and another study showed that weight loss induced by laparoscopic adjustable gastric banding is also associated with decreased hepatic TSK expression [11]. At present, there is a lack of research on circulating TSK levels in subjects with obesity and NAFLD, and there are no population studies to analyze the association between TSK levels and detailed metabolic traits. It would be of great significance to clarify the role of TSK in human metabolic disease.

Thus, we conducted a study on serum TSK levels in subjects with obesity and lean controls. Furthermore, we explored single nucleotide polymorphisms (SNPs), which were associated with serum TSK levels in a Han Chinese population, and analyzed the association between the SNPs and metabolic traits.

## Materials and methods

### Study design and participants

This cross-sectional study explored the association between serum TSK levels and obesity in 144 people in China and the association between SNP and serum TSK levels, as well as SNP and metabolic traits, in a Chinese population of 11,022 individuals.

The individuals enrolled in the current study were selected from a prospective population-based study (*n* = 18,033) in Shanghai designed to investigate the occurrence of various metabolic diseases [12]. Participants with a medical history of surgery, trauma, pregnancy, cancer, other chronic severe liver diseases and kidney disease, psychiatric disturbance, insufficient or unavailable data on laboratory measurements, or a history of drug use were excluded from this study. A total of 11,022 remaining individuals were selected for genotype analysis. Individuals without detailed drug use history and diagnosed as new-onset diabetes mellitus were also excluded. A total of 914 participants were included for further evaluation. According to the clinical diagnosis for obesity and overweight, 223 individuals with available fresh serum (BMI ≥ 28 kg/m^2^ or BMI < 24 kg/m^2^) were selected using simple random sampling. After being grouped for age, a total of 144 individuals were selected to assess their levels of serum TSK, including 103 individuals in the obesity group and 41 in the lean group (Supplemental Figure 1). Basic anthropometric data were collected, including age, gender, height, weight, and waist circumference (WC). The criteria for obesity in China is defined as BMI ≥ 28 kg/m^2^ [13]. Body fat (%) was measured using a TBF-410 Tanita Body Composition Analyzer (Tanita, Tokyo, Japan). Abdominal scans were performed at the umbilicus level, between the lumbar spinal vertebrae L4 and L5 (Philips magnetic resonance imaging system, Netherlands). Visceral fat area (VFA) and subcutaneous fat area (SFA) (cm^2^) were assessed by 3 radiologists, using Slice-O-Matic software (version 4.2, Tom Vision, Canada). Individuals received an oral glucose tolerance test (OGTT), and venous blood samples were collected at 0, 30 and 120 min to measure plasma glucose and insulin levels by radioimmunoassay (Linco Research, St Charles, MO, USA). Insulin secretion and sensitivity were estimated by homeostasis model assessment [14], the Stumvoll Index [15], and Gutt Index [16]. Ethical approval was obtained from the Ethics Committee of Shanghai Jiao Tong University Affiliated Sixth People’s Hospital, in accordance with the Declaration of Helsinki II. All subjects provided written, informed consent.

### Serum TSK measurement

Peripheral blood samples were collected from the vein, and serum was isolated and stored at – 80 ℃ until use. Serum TSK levels were assessed using enzyme-linked immunosorbent assay (ELISA) using human Tsukushi/TSK ELISA kits (CODE: ELH-TSKU-1; RayBiotech, Inc., Georgia, United States). The protocol was provided in the kit.

### Genotyping and quality control

The SNPs in the TSKU gene region (30 kb upstream and downstream) were genotyped using the Infinium Multi-Ethnic Global BeadChip, and Infinium Asian Screening Array, optimized for East Asian populations (Illumina, Inc., San Diego, CA, USA). The genotype data were imputed according to 504 East-Asian subjects from 1000 Genomes [17, 18]. The imputation quality was evaluated by rsq values (estimated *r*^2^, specific to each SNP), and all SNPs had rsq values ≥ 0.5. The rsq values of rs11236956 were 0.88 and 0.82 for each genotype array. All of the SNPs passed quality control with call rates ≥ 95% and Hardy Weinberg balance (*P* > 0.05). Rs11236956 was detected in 11,021/11,022 subjects.

### Statistical analysis

SAS (version 9.2; SAS Institute, Cary, NC, USA) and Plink (http://pngu.mgh.harvard.edu/~purcell/plink/) were used to perform the analysis. The skewed distribution traits were log10-transformed to fit normal distribution. *Χ*^2^ tests were used to analyze differences in frequency. *T*-test and one-way analysis of variance were used to analyze the trait difference between groups. Serum TSK levels were shown as the median and interquartile range, and the significance of differences in TSK levels was calculated using the Mann–Whitney *U* test. Serum TSK levels were transformed to hierarchical data in tertiles of the total population. Trend tests, under logistic regression and multiple linear regression were used to analyze the association between grouped or continuous traits and TSK levels, adjusting for age and gender. Haploview [19] was used to analyze linkage disequilibrium among the SNPs. Logistic regression and multiple linear regression were also used to calculate the effect of SNPs on grouped or continuous traits under the additive genetic model. A two-tailed *P* value of 0.05 was considered significant.

Power calculations were performed using Quanto software (http://biostats.usc.edu/Quanto.html). With a sample size of 11,022, the SNP rs11236956 (minor allele frequency: 0.4133) had more than 90% power to analyze the association with continuous traits (set as mean value ± stand deviation = 10 ± 5) at an effect size (*β* value) of 0.2. A two-tailed *P* value was set at 0.05. The study also had more than 95% power to analyze the association in case–control studies at an effect size (odds ratio) of 1.1 with rs11236956 (minor allele frequency: 0.4133) at 0.05 significance.

## Results

### Clinical characteristics

We recruited 103 obese individuals and 41 lean controls into the study. Their clinical and anthropometric features are shown in Table 1. The obesity group had higher BMI, WC, VFA, SFA, fasting plasma glucose (FPG), glycated hemoglobin (HbA1c), total cholesterol (TC) and total triglyceride (TG). The two groups showed no difference in age, liver function, low-density lipoprotein-cholesterol (LDL), high-density lipoprotein-cholesterol (HDL), insulin, or FFAs levels in plasma. There was no significant difference between the two groups in their 30-min plasma glucose and 2-h plasma glucose levels after OGTT.

**Table 1:** Metabolic traits of subjects

	Obesity group (*n* = 103)	Control (*n* = 41)	*P* value
Non-alcoholic fatty liver disease (%)	70.88%	0%	–
Gender (male%)	32.90%	60.61%	–
Age (year)	63.09 ± 3.93	63.46 ± 1.41	0.4041
Body mass index (kg/m^2^)	29.82 ± 3.54	22.07 ± 3.49	< 0.0001
Waist circumference (cm)	89.58 ± 9.89	80.69 ± 8.34	< 0.0001
Visceral fat area (cm^2^)	136.07 ± 55.86	107.99 ± 49.95	0.0091
Subcutaneous fat area (cm^2^)	205.23 ± 70.30	103.02 ± 46.71	< 0.0001
Fast plasma glucose (mmol/L)	6.26 ± 1.30	5.83 ± 0.86	0.0411
30 min plasma glucose after OGTT (mmol/L)	10.10 ± 1.84	9.88 ± 2.22	0.5311
2 h plasma glucose after OGTT (mmol/L)	9.89 ± 4.32	9.16 ± 4.56	0.3494
Fast plasma insulin (mU/L)	13.70 ± 29.94	9.10 ± 27.98	0.3763
30 min insulin after OGTT (mmol/L)	58.04 ± 46.27	46.96 ± 40.38	0.1606
2 h plasma insulin after OGTT (mmol/L)	68.61 ± 63.40	49.58 ± 64.0	0.0929
HbA1c (%)	5.99 ± 0.98	5.67 ± 0.58	0.0399
Total cholesterol (mmol/L)	4.51 ± 0.46	4.31 ± 0.46	0.0183
Total triglyceride (mmol/L)	1.14 ± 0.36	0.97 ± 0.32	0.0062
Low density lipoprotein-cholesterol (mmol/L)	2.59 ± 0.44	2.49 ± 0.45	0.1929
High density lipoprotein-cholesterol (mmol/L)	1.31 ± 0.28	1.27 ± 0.27	0.4256
Alanine aminotransferase (U/L)	18.15 ± 7.34	16.83 ± 6.48	0.2917
Aspartate aminotransferase (U/L)	23.17 ± 5.54	23.56 ± 5.92	0.6938
γ-glutamyltransferase (U)	23.94 ± 15.39	28.91 ± 14.73	0.0661
Free fatty acids (umol/L)	606.37 ± 208.81	550.39 ± 245.49	0.1609

Data are shown as mean ± standard deviation or *N*%

*OGTT* oral glucose tolerance test

### Serum TSK levels

**Fig. 1:**
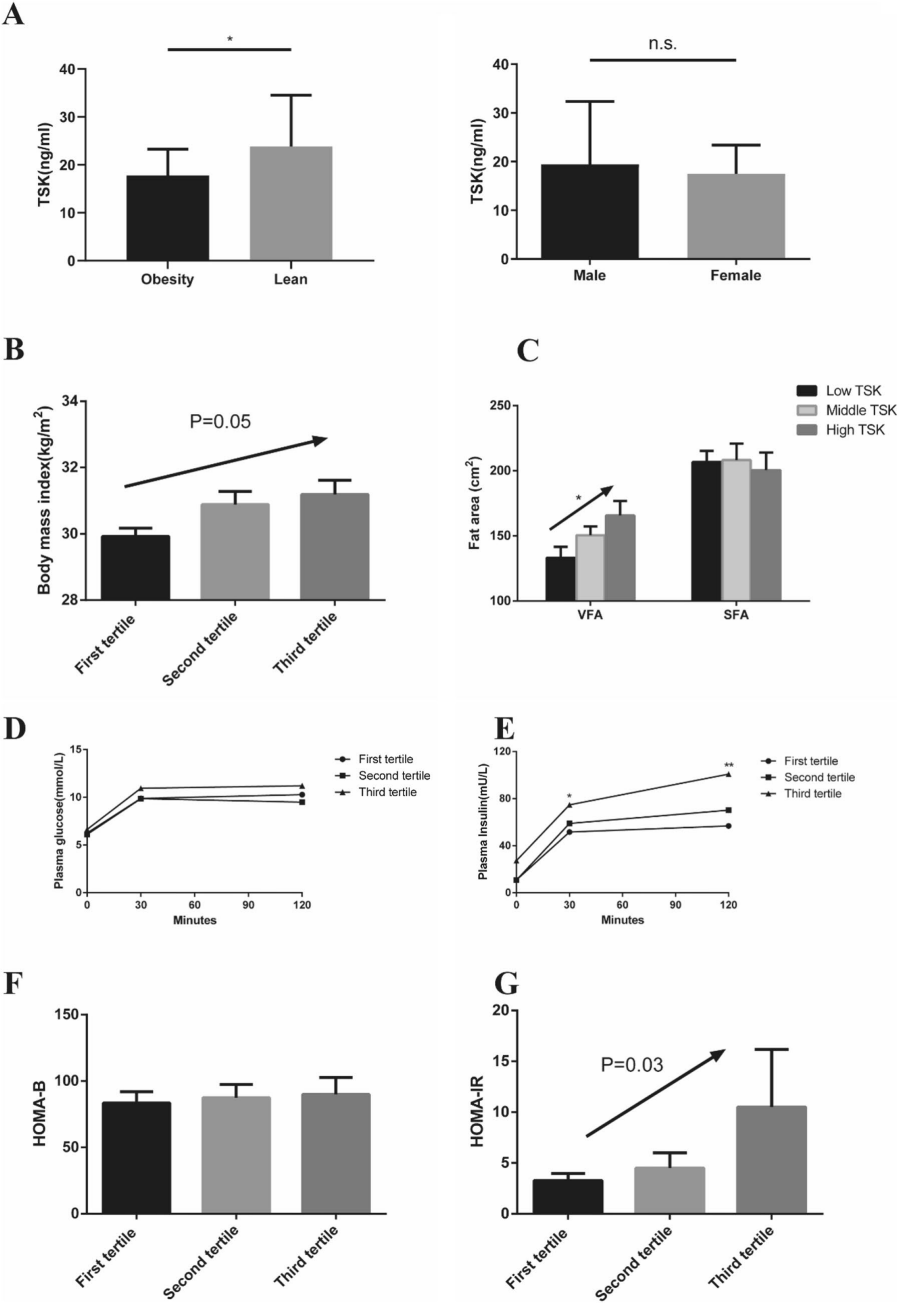
The association between serum TSK level and metabolic traits. **a** Comparison of serum TSK level between the obesity group (*N* = 103) and the lean group (*N* = 41), and between males (*N* = 48) and females (*N* = 96) in the total population. Data are shown as median and interquartile ranges in histograms. Significance of differences was calculated using the non-parametric test. **b** In obese subjects, the association between serum TSK level (in tertiles) with BMI (*P* = 0.05). **c** In obesity subjects, the association between serum TSK level (in tertiles) with VFA (*β* ± SE: 12.15 ± 5.94, *P* = 0.011) and SFA. **d**, **e** In the obesity subgroup, the curve of plasma glucose and insulin after OGTT in different serum TSK level groups. In the comparison, subjects with a low serum TSK level were used as a reference. **f**, **g** In the obesity subgroup, the association between serum TSK level (in tertiles) with HOMA-β and HOMA-IR (*P* = 0.03). For TSK levels in the obesity subgroup, first tertile: *N* = 35, second tertile: *N* = 40, third tertile: *N* = 28. OGTT: oral glucose tolerance test. HOMA-β: homeostatic model assessment for *β* cell. HOMA-IR: homeostatic model assessment for insulin resistance index. Data are shown as mean ± SEM in histogram. Significance of differences was calculated using the Student’s *t*-test. *P < 0.05, **P < 0.01, ***P < 0.001

The range of plasma TSK was 0.21–396.85 ng/ml in our population, with an average concentration of 28.62 ng/ml (median: 27.82; interquartile range: 21.33, 38.14). The results showed no difference between males and females, or between those under 60 and over (Fig. 1a, Supplemental Figure 2A). Surprisingly, we found that the plasma TSK level in the obesity group (median and interquartile range: 17.78: 12.07–23.28) was lower than that in the lean group (23.81:12.54–34.56, *P* < 0.05) (Fig. 1a). As is well-known, NAFLD is strongly associated with overweight or obesity. To avoid the influence of NAFLD on the association of obesity and TSK, we compared the TSK levels between subjects with NAFLD and without NAFLD and found no significant difference between the two groups (Supplemental Figure 2B). However, obese subjects with or without NAFLD had similar TSK levels, but both were lower than that of the lean subjects (*P* < 0.05, Supplemental Figure 2C).

**Fig. 2:**
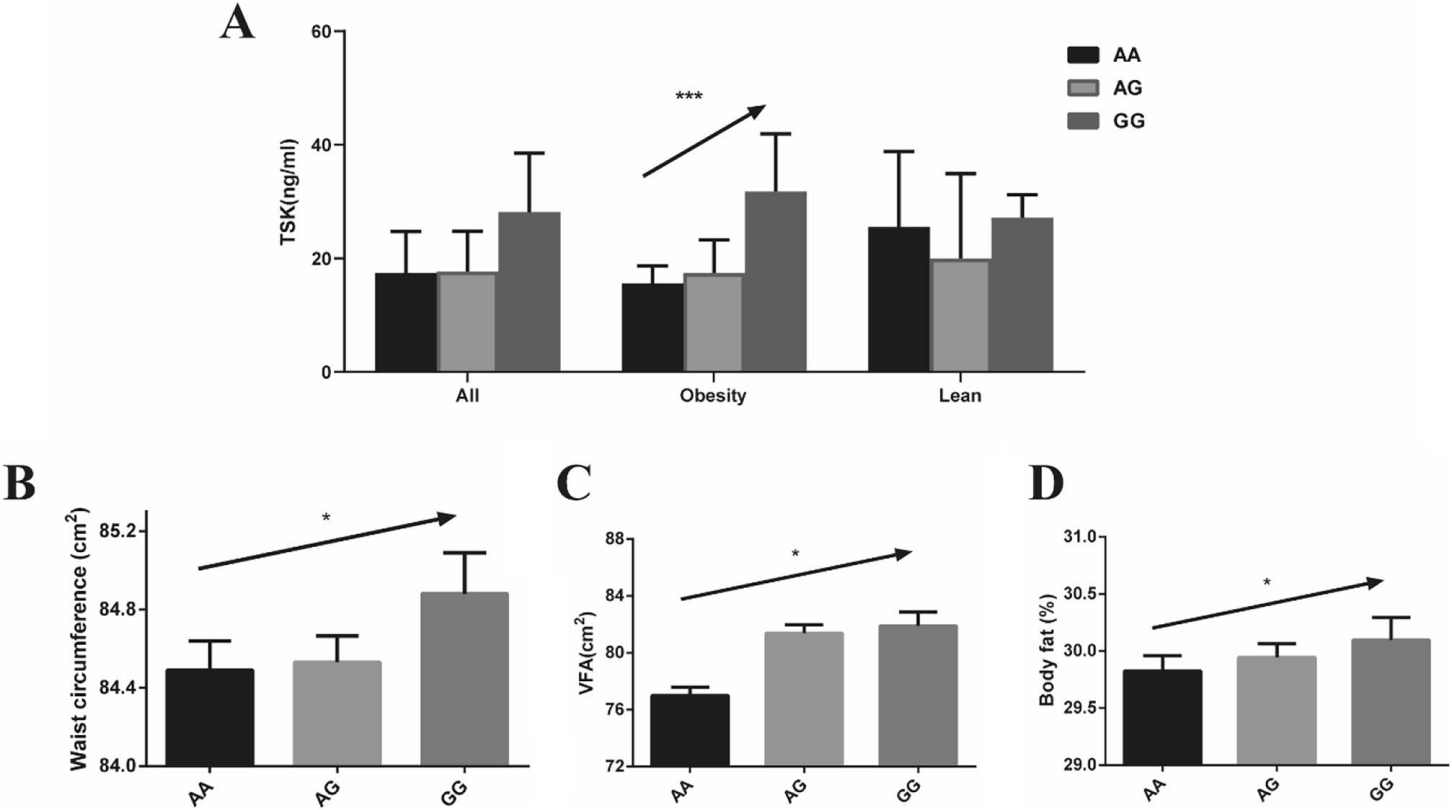
Association between rs11236956 and serum TSK level. **a** Association between rs11236956 genotype with serum TSK level in total population (AA group: *N* = 29; AG group: *N* = 59; GG group: *N* = 23), in subjects with obesity (AA group: *N* = 19; AG group: *N* = 44; GG group: *N* = 18) and in lean controls (AA group: *N* = 10; AG group: *N* = 15; GG group: *N* = 5). Data are shown as a median and interquartile range in the histogram. Significance of differences was calculated using the non-parametric test. **b**–**d** Association between rs11236956 genotype with waist circumference, VFA and body fat in subjects with obesity. Data are shown as mean ± SEM in the histogram. Significance of differences was calculated using the Student’s *t*-test. *P < 0.05, **P < 0.01, ***P < 0.001

### Association of plasma TSK level with metabolic traits in the obesity subgroup

The total population was divided into tertiles, according to their TSK levels. In the obesity group, there were 35, 40 and 28 subjects assigned in the first, middle and high tertiles. We constructed a multiple linear regression model to evaluate the dose–response association of TSK with metabolic traits, after adjusting for age and gender. In the total population, metabolic traits were not statistically associated with TSK level, except for the 2-h plasma insulin level after OGTT (Supplemental Table 1). Considering the lower mean TSK level of the obesity group, we hypothesized that BMI might be a potential confounding variable. We performed tests for trends in subgroups based on BMI, and found that, in the obesity group, serum TSK level was associated with BMI (*β* ± SE: 0.63 ± 0.31, *P* = 0.049), VFA (*β* ± SE: 12.15 ± 5.94, *P* = 0.011), 30-min insulin after OGTT (*β* ± SE: 11.70 ± 5.82, *P* = 0.045), 2-h insulin after OGTT (*β* ± SE: 21.14 ± 8.02, *P* = 0.009), and homeostatic model assessment for insulin resistance (HOMA-IR) index (*β* ± SE: 3.57 ± 1.61, *P* = 0.027) (Table 2 and Fig. 1b–g). However, we did not find any association between TSK level and metabolic traits in the lean group (Supplemental Table 2). These results suggest that the serum level of TSK is associated with the severity of obesity.

**Table 2:** Association of serum TSK level with metabolic traits in obesity group

	Low TSK (*n* = 35)	Middle TSK (*n* = 40)	High TSK (*n* = 28)	*β* ± SE	*P* value
Body mass index (kg/m^2^)	29.92 ± 0.25	30.88 ± 0.40	31.18 ± 0.46	0.63 ± 0.31	**0.048**
Subcutaneous fat area (cm^2^)	206.77 ± 8.59	208.23 ± 12.75	200.43 ± 13.68	7.90 ± 0.02	0.948
Visceral fat area (cm^2^)	133.19 ± 8.42	150.48 ± 6.84	156.95 ± 12.64	12.16 ± 5.94	**0.011**
Fast plasma glucose (mmol/L)	6.25 ± 0.25	6.11 ± 0.15	6.63 ± 0.28	0.18 ± 0.17	0.286
30 min plasma glucose after OGTT (mmol/L)	9.89 ± 0.31	9.85 ± 0.32	10.95 ± 0.36	0.48 ± 0.55	0.051
2 h plasma glucose after OGTT (mmol/L)	10.29 ± 0.86	9.49 ± 0.59	11.21 ± 0.80	0.37 ± 0.02	0.336
Fast plasma insulin (mU/L)	10.95 ± 1.87	10.81 ± 1.28	27.38 ± 11.82	7.99 ± 4.27	0.064
30 min insulin after OGTT (mmol/L)	51.65 ± 4.69	58.98 ± 7.77	74.77 ± 9.86	11.70 ± 5.82	**0.045**
2 h plasma insulin after OGTT (mmol/L)	56.85 ± 5.31	70.23 ± 10.08	100.82 ± 14.43	21.14 ± 8.02	**0.009**
Total cholesterol (mmol/L)	4.35 ± 0.09	4.59 ± 0.07	4.40 ± 0.09	0.04 ± 0.06	0.478
Total triglyceride (mmol/L)	1.11 ± 0.06	1.14 ± 0.06	1.19 ± 0.07	0.04 ± 0.05	0.413
Low density lipoprotein-cholesterol (mmol/L)	2.50 ± 0.08	2.70 ± 0.08	2.59 ± 0.08	0.05 ± 0.06	0.361
High density lipoprotein-cholesterol (mmol/L)	1.26 ± 0.04	1.26 ± 0.04	1.24 ± 0.05	− 0.01 ± 0.03	0.651
HOMA-B	83.39 ± 8.56	87.47 ± 9.98	139.97 ± 46.90	27.03 ± 17.56	0.127
HOMA-IR	3.27 ± 0.71	3.01 ± 0.38	10.51 ± 4.66	3.57 ± 1.61	**0.027**
γ-glutamyltransferase (U)	23.71 ± 1.69	26.25 ± 2.52	28.14 ± 3.61	2.09 ± 1.97	0.279
Alanine aminotransferase (U/L)	18.57 ± 1.31	19.48 ± 1.05	20.14 ± 1.44	0.73 ± 0.92	0.429
Aspartate aminotransferase (U/L)	23.54 ± 1.14	23.45 ± 0.79	24.46 ± 1.14	0.40 ± 0.74	0.594

Relationship between TSK level and metabolic traits in obesity population. Metabolic traits were shown by mean ± sem. Analysis was performed on trend test under multilinear regression. Results were shown as *β* ± SE. *P* < 0.05 were in bold

### SNPs in the TSKU gene region with serum TSK level in obesity

The level of TSK was lower in obese individuals; however, it was positively associated with BMI in the obesity subgroup. To determine the association between TSK and metabolic traits, we performed microarray analysis to explore *TSKU*-related SNPs in the obesity group, which attenuated the confounding effect of BMI (Supplemental Figure 3). Three SNPs (rs11236956, rs1660579, and rs11236955) in high-linkage disequilibrium (*D*ʹ = 1, *r*^2^ > 0.99) were associated with serum TSK level in the obese group, after adjusting for age and gender (*β* 95% CI 0.17, 0.07–0.26; *P* = 0.0007) (Fig. 2a, Supplemental Table 3). We chose rs11236956 as a tag SNP, with reference allele A and alternate allele G. It has been reported that the East-Asian population has the highest minor allele frequency (*G* = 0.4623), according to 1000 Genomes (African, *G* = 0.2761; European, *G* = 0.2366; South Asian, *G* = 0.2330; American, *G* = 0.2490) [17, 18]. Similarly, in our total population of 11,021 individuals, the frequency of G allele for rs11236956 was 0.4133 (Supplemental Table 4). The metabolic traits of subjects carrying different rs11236956 genotypes are shown in Supplemental Table 4.

### Association of rs11236956 with metabolic traits in a Chinese population

We constructed an additive genetic model under multiple linear and logistic regressions, adjusted for age and gender (Table 3). In the total population, rs11236956-G was associated with WC (*β* = 0.49; 95% CI 0.09–0.89; *P* = 0.0160), VFA (*β* = 4.34; 95% CI 0.22–8.46; *P* = 0.0391), body fat (*β* = 0.16; 95% CI 0.00–0.31; *P* = 0.0440), 30-min plasma glucose after OGTT (*β* = 0.10; 95% CI 0.04–0.16; *P* = 0.0017), 2-h plasma glucose after OGTT (*β* = 0.20; 95% CI 0.09–0.31); *P* = 0.0004), GAUC (*β* = 0.26; 95% CI 0.12–0.44; *P* = 0.0002), HbA1c (*β* = 0.04; 95% CI 0.01–0.06; *P* = 0.0067), and 2-h plasma insulin after OGTT (*β* = 1.40; 95% CI 0.06–2.74; *P* = 0.0409) (Table 3). In addition, we calculated the Stumvoll index to evaluate the ability of pancreatic *β* cells to secrete insulin. Subjects with the *G* allele had weaker ability to secrete insulin, from Stumvoll 1st index (*β* = − 12.85; 95% CI − 19.78–5.93; *P* = 0.0003), and Stumvoll 2nd index (*β* = − 4.36; 95% CI − 6.20–2.52; *P* = 3.52 × 10^–6^). Moreover, subjects with the *G* allele had a higher FFA (*β* = 11.30; 95% CI 5.04–17.56; *P* = 0.0004) and γ-GT level (*β* = 2.16; 95% CI 1.02–3.30; *P* = 0.0002). In the obese group (*n* = 1879), the correlation between some metabolic traits and rs11236956 still existed (Table 3, Fig. 2b-d). However, compared with the whole population, the statistical significance was weak, due to the limitation of sample size.

**Table 3:** Association between rs11236956 with metabolic traits in a Chinese population

Traits	ALL (*n* = 11,022)	*P* value	Obesity (*n* = 1879)	*P* value
	*β* or OR 95% CI		*β* or OR 95% CI	
Body mass index (kg/m^2^)	0.077 (− 0.01, 0.16)	0.0784	0.01 (− 0.08, 0.10)	0.7995
Waist circumference (cm^2^)	0.49 (0.09, 0.89)	**0.0160**	0.59 (− 0.14, 1.32)	0.1124
Visceral fat area (cm^2^)	4.34 (0.22, 8.46)	**0.0391**	5.65 (1.03, 10.27)	**0.0168**
Subcutaneous fat area (cm^2^)	2.74 (− 3.57, 9.05)	0.3948	− 2.24 (− 6.94, 2.45)	0.3495
Bodyfat (%)	0.16 (0.00, 0.31)	**0.0440**	0.08 (− 0.07, 0.22)	0.2986
Fatmass (kg)	0.18 (− 0.02, 0.37)	0.0752	0.13 (− 0.12, 0.38)	0.3135
Nonfatmass (kg)	0.02 (− 0.22, 0.26)	0.875	0.23 (− 0.33, 0.79)	0.4217
Type 2 diabetes	1.02 (1.00, 1.05)	0.0518	1.05 (1.01, 1.10)	**0.0395**
Fast plasma glucose (mmol/L)	0.04 (− 0.00, 0.08)	0.0728	0.03 (− 0.04, 0.10)	0.4144
30 min plasma glucose (mmol/L)	0.10 (0.04, 0.16)	**0.0017**	0.17 (0.07, 0.28)	**0.0015**
2 h plasma glucose after (mmol/L)	0.20 (0.09, 0.31)	**0.0004**	0.22 (0.03, 0.41)	**0.0252**
Glucose area under curve	0.26 (0.12, 0.40)	**0.0002**	0.34 (0.11, 0.58)	**0.0046**
HbA1c (%)	0.04 (0.01, 0.06)	**0.0067**	0.01 (− 0.03, 0.05)	0.6430
Fast plasma insulin (mU/L)	− 0.07 (− 0.59, 0.44)	0.7857	− 0.77 (− 1.94, 0.40)	0.1985
30 min insulin (mmol/L)	− 0.32 (− 1.54, 0.90)	0.6088	− 0.59 (− 2.99, 1.82)	0.6332
2 h plasma insulin after (mmol/L)	1.40 (0.06, 2.74)	**0.0409**	1.98 (− 0.70, 4.66)	0.1474
Insulin area under curve	0.62 (− 1.35, 2.58)	0.5393	0.15 (− 3.85, 4.16)	0.9401
Stumvoll 1st index	− 12.85 (− 19.78, − 5.93)	**0.0003**	− 19.07 (− 31.92, − 6.21)	**0.0037**
Stumvoll 2nd index	− 4.36 (− 6.20, − 2.52)	**3.52 × 10**^**–6**^	− 4.5 3(− 7.89, − 1.16)	**0.0085**
Gutt index	− 0.43 (− 0.89, 0.02)	0.0640	− 0.65 (− 1.48, 0.18)	0.1239
Free fatty acid (mmol/L)	11.30 (5.04, 17.56)	**0.0004**	2.80 (− 7.02, 12.61)	0.5767
Total cholesterol (mmol/L)	0.01 (− 0.02, 0.03)	0.6148	0.04 (− 0.01, 0.08)	0.0984
Total triglyceride (mmol/L)	0.04 (− 0.00, 0.08)	0.0650	0.06 (− 0.02, 0.14)	0.1655
High density lipoprotein-cholesterol (mmol/L)	− 0.01(− 0.01, 0.00)	0.3384	0.00 (− 0.01, 0.01)	0.8544
Low density lipoprotein-cholesterol (mmol/L)	0.01 (− 0.02, 0.03)	0.5535	0.03 (− 0.01, 0.06)	0.0999
γ-glutamyltransferase (U)	2.16 (1.02, 3.30)	**0.0002**	2.14 (0.26, 4.03)	**0.0260**

*CI* confidence interval

*P*<0.05 were showed in bold

## Discussion

**Fig. 3:**
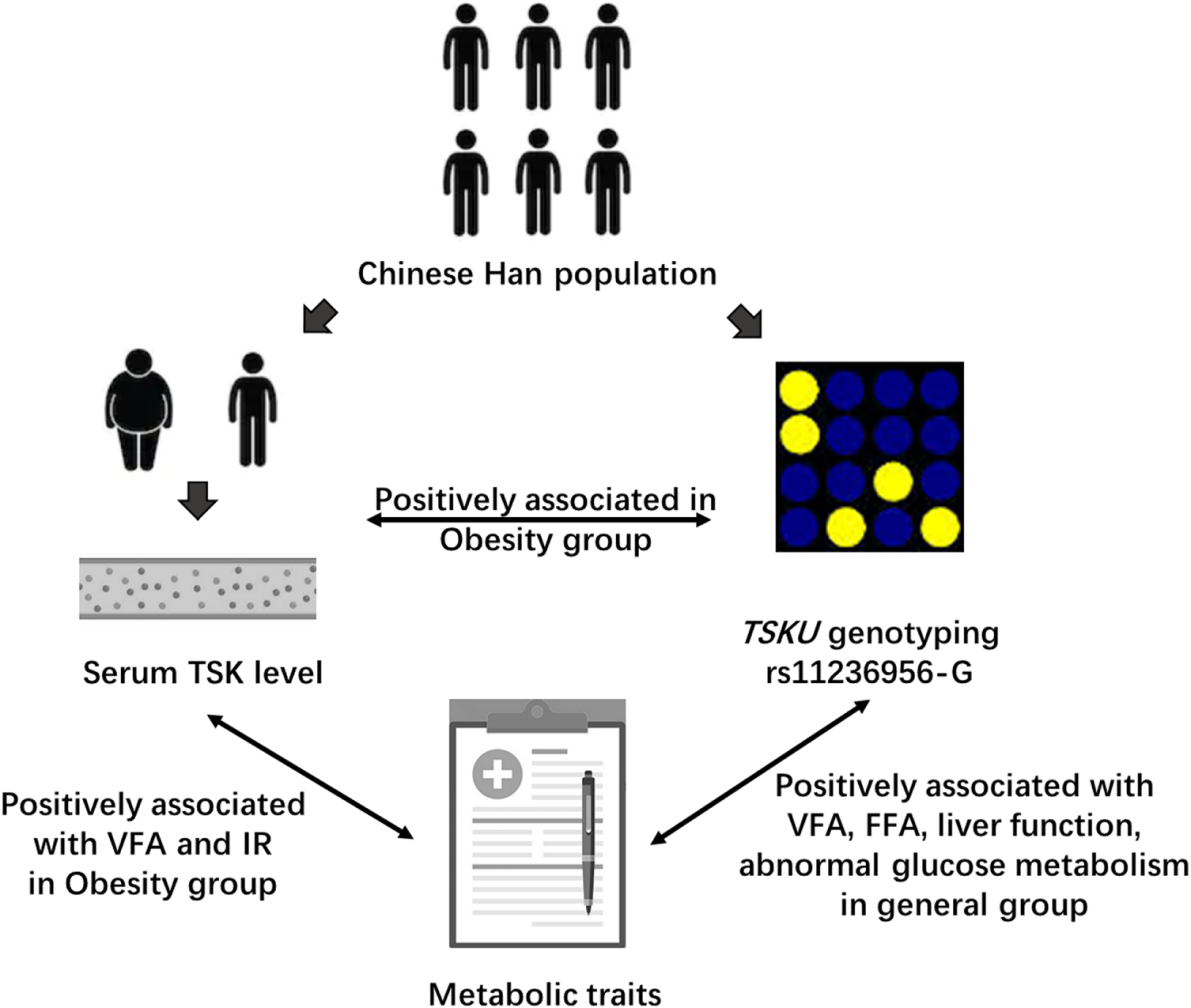
Association of serum TSK level, *TSKU* genotype and metabolic traits. Obesity subjects with genotype of rs11236956-G had high serum TSK level, they also had high visceral fat accumulated and insulin resistance. In general population, obesity subjects with genotype of rs11236956-G also had higher visceral fat accumulated, free fat acid, poor liver function and abnormal glucose metabolism. *VFA* visceral fat area, *IR* insulin resistance, *FFA* free fatty acid

Our study showed that the level of TSK was correlated with the severity of obesity and with glucose homeostasis. We also identified that rs11236956, a novel SNP located in *TSKU*, was associated with serum TSK level. Further analysis showed that subjects with risk allele G of rs11236956 had higher body fat, and were more likely to develop an excessive accumulation of white adipose tissue in the abdominal cavity, which caused an increase in metabolic disorders (Fig. 3).

To the best of our knowledge, our results are the first to assess the serum TSK concentration in a community-based population. In previous studies, TSK was defined as a hepatokine, induced by obesity and NAFLD [8, 9]. It has been reported that inflammation and endoplasmic reticular stress robustly promoted the release of TSK, especially in acetaminophen-induced acute liver failure [9]. However, in our study, the level of TSK was lower in the obesity group than that in the control but positively correlated with BMI in the obesity subgroup. We suspected that, collectively, the TSK level was regulated in a compensatory manner, although a positive correlation with BMI was identified in our obesity subgroup. With BMI increasing, the apparent bidirectional trends in TSK level suggest that the liver could regulate the secretion of TSK by a compensation mechanism. In the early stages of obesity, due to mild hepatic steatosis, the expression of TSK decreased as part of a negative feedback mechanism emerging from the liver. Conversely, severe liver damage resulted in extremely high levels of circulating TSK, occurring in patients that died or received a liver transplant [9]. It is possible that the massive release of TSK is apparent only in the case of extreme liver dysfunction.

It is known that genetic background plays an important role in the progression of the disease [20]. We hypothesized that the genetic background may influence the ability to compensate for TSK expression in moderate metabolic disorders. Thus, we screened the *TSKU* gene region and identified rs11236956 as a novel variant associated with serum TSK level in subjects with obesity. Individuals with risk allele G had a higher TSK level. Moreover, we explored the association of rs11236956 with metabolic traits in a Chinese population (*n* = 11,021). Consistently, rs11236956-G was confirmed to be associated with higher WC, body fat and VFA. Furthermore, rs11236956 was also associated with glucose metabolic homeostasis, such as plasma glucose after OGTT and insulin secretion index (1st and 2nd Stumvoll Indices), which suggests that rs11236956-G allele carriers (who tended to have a higher TSK level) had a weaker ability to secrete insulin. It has been reported, in epigenomic annotations, that rs11236956 is located in a transcriptional regulatory region for adipose tissue and liver (Supplemental Figure 4) (T2DKP Knowledgebase: http://www.type2diabetesgenetics.org/). We also found that rs11236956-G was positively associated with FFAs and γ-GT, and FFAs may be the “bridge” linking excessive visceral fat and adipose tissue.

There are some limitations to the present study. First, the sample size of the study was small because we could not obtain sufficient samples from individuals who were obese but did not have NAFLD to explore the correlation between TSK level and BMI excluding NAFLD status. Second, our study was cross-sectional; thus, we could not evaluate the effect of TSK on disease progression. Third, the variant rs11236956 was located in a non-coding region, and the potential relationship among the variant, obesity, and related metabolic traits warrants further investigation. Lastly, because we only investigated the relationship in a Han Chinese population, the results should be confirmed in other ethnicities. Nonetheless, our findings provide novel information and imply the potential involvement of TSK in metabolic disorders.

## Supplementary Information

Below is the link to the electronic supplementary material.

Supplementary file1 (DOCX 1155 kb)

## Data Availability

All data relevant to the study are included in the article or uploaded as supplemental information.
