# Supplementary material for: Tsukushi and TSKU genotype in obesity and related metabolic disorders
Source: J Endocrinol Invest. 2021 Apr 15;44(12):2645–54. doi: 10.1007/s40618-021-01572-x (PMC8572186; doi:10.1007/s40618-021-01572-x)
Supplement: Supplementary file 1 — Supplementary file1 (DOCX 1155 kb) [file 40618_2021_1572_MOESM1_ESM.docx]

Supplemental Table 1. Association of serum TSK level with metabolic traits in total population.

|  | Low TSK (n=47) | Middle TSK (n=48) | High TSK (n=49) | Beta±se | *P* value |
| --- | --- | --- | --- | --- | --- |
| Body mass index (kg/m^2^) | 26.89±0.55 | 28.53±0.55 | 26.30±0.65 | -0.16±0.37 | 0.669 |
| Subcutaneous fat area(cm^2^) | 2.24±0.03 | 2.23±0.03 | 2.08±0.04 | 8.47±7.27 | 0.246 |
| Visceral fat area(cm^2^) | 2.05±0.03 | 2.13±0.02 | 2.00±0.04 | -0.57±6.24 | 0.927 |
| Fast plasma glucose(mmol/L) | 6.11±0.19 | 6.08±0.13 | 6.17±0.19 | -0.07±0.12 | 0.570 |
| 30 minutes plasma glucose after OGTT(mmol/L) | 9.89±0.27 | 9.80±0.28 | 10.38±0.31 | -0.27±0.20 | 0.186 |
| 2 hours plasma glucose after OGTT(mmol/L) | 9.43±0.68 | 9.70±0.65 | 9.80±0.58 | -0.24±0.46 | 0.603 |
| Fast plasma insulin(mU/L) | 9.12±1.47 | 9.88±1.14 | 17.27±6.91 | -4.75±3.03 | 0.120 |
| 30 minutes insulin after OGTT(mmol/L) | 47.33±3.86 | 55.94±7.20 | 59.52±7.47 | -7.51±4.58 | 0.103 |
| 2 hours plasma insulin after OGTT(mmol/L) | 49.56±4.50 | 62.59±8.77 | 74.16±12.28 | -14.30±6.50 | **0.029** |
| Total cholesterol(mmol/L) | 4.37±0.08 | 4.56±0.06 | 4.42±0.06 | -0.04±0.05 | 0.362 |
| Total triglyceride(mmol/L) | 2.49±0.07 | 2.67±0.07 | 2.53±0.06 | -0.03±0.05 | 0.507 |
| Low density lipoprotein-cholesterol(mmol/L) | 1.10±0.05 | 1.11±0.05 | 1.05±0.05 | 0.01±0.04 | 0.819 |
| High density lipoprotein-cholesterol(mmol/L) | 1.31±0.04 | 1.27±0.04 | 1.30±0.04 | 0.00±0.03 | 0.985 |
| HOMA-B | 71.21±7.15 | 80.63±8.93 | 96.57±27.62 | -15.03±12.61 | 0.236 |
| HOMA-IR | 2.68±0.54 | 2.73±0.34 | 6.41±3.28 | -2.19±1.42 | 0.124 |
| γ-glutamyltransferase(U) | 1.34±0.03 | 1.34±0.03 | 1.36±0.03 | -0.66±1.58 | 0.679 |
| Alanine aminotransferase(U/L) | 1.20±0.03 | 1.24±0.02 | 1.21±0.02 | -0.18±0.74 | 0.808 |
| Aspartate aminotransferase(U/L) | 1.34±0.02 | 1.36±0.01 | 1.36±0.02 | -0.47±0.59 | 0.429 |

The total population was divided into three groups according to the TSK level tertile. Metabolic traits were shown by mean ± se. Analysis were performed on trend test under multilinear regression. Results were shown as beta ± sem. *P* <0.05 were in bold. OGTT: Oral glucose tolerance test.

Supplemental Table 2. Association of serum TSK level with metabolic traits in lean group

|  | First tertile of TSK (n=12) | Second tertile of TSK (n=8) | Third tertile of TSK (n=21) | Beta±se | *P* value |
| --- | --- | --- | --- | --- | --- |
| Body mass index (kg/m^2^) | 19.98±0.43 | 20.83±6.04 | 20.57±0.26 | -0.01±0.00 | 0.281 |
| Subcutaneous fat area(cm^2^) | 82.87±8.59 | 83.88±36.82 | 75.40±7.23 | 2.58±6.34 | 0.687 |
| Visceral fat area(cm^2^) | 65.30±11.76 | 95.00±42.30 | 68.43±7.21 | 4.91±6.90 | 0.482 |
| Fast plasma glucose(mmol/L) | 5.70±0.17 | 6.01±1.30 | 5.55±0.17 | 0.11±0.13 | 0.411 |
| 30 minutes plasma glucose after OGTT(mmol/L) | 9.89±0.56 | 9.56±2.52 | 9.63±0.49 | 0.05±0.36 | 0.897 |
| 2 hours plasma glucose after OGTT(mmol/L) | 6.94±0.46 | 10.79±7.68 | 7.92±0.67 | -0.02±0.03 | 0.617 |
| Fast plasma insulin(mU/L) | 3.80±0.46 | 4.59±2.15 | 3.78±0.49 | 0.03±0.38 | 0.939 |
| 30 minutes insulin after OGTT(mmol/L) | 34.73±5.12 | 40.81±52.13 | 39.19±4.77 | -0.02±0.06 | 0.680 |
| 2 hours plasma insulin after OGTT(mmol/L) | 28.31±4.71 | 24.42±12.70 | 38.61±13.67 | -0.04±0.06 | 0.461 |
| Total cholesterol(mmol/L) | 4.42±0.17 | 4.45±0.64 | 4.43±0.09 | 0.00±0.01 | 0.539 |
| Total triglyceride(mmol/L) | 1.07±0.10 | 0.98±0.63 | 0.86±0.06 | 0.09±0.05 | 0.103 |
| Low density lipoprotein-cholesterol(mmol/L) | 2.44±0.11 | 2.56±0.27 | 2.45±0.09 | 0.00±0.02 | 0.986 |
| High density lipoprotein-cholesterol(mmol/L) | 1.46±0.08 | 1.36±0.57 | 1.40±0.06 | 0.11±0.08 | 0.181 |
| HOMA-B | 35.69±4.88 | 41.54±28.20 | 38.72±4.31 | -0.01±0.01 | 0.680 |
| HOMA-IR | 0.97±0.12 | 1.21±0.72 | 0.95±0.13 | 0.02±0.10 | 0.829 |
| γ-glutamyltransferase(U) | 26.58±6.28 | 22.88±14.23 | 24.57±2.86 | 0.74±2.95 | 0.803 |
| Alanine aminotransferase(U/L) | 13.33±1.49 | 13.88±5.35 | 13.67±0.97 | 0.00±0.02 | 0.892 |
| Aspartate aminotransferase(U/L) | 20.33±0.94 | 23.38±7.80 | 22.71±1.28 | -0.02±0.02 | 0.341 |

Relationship between TSK level and metabolic traits in lean population. Metabolic traits were shown by mean ± sem. Analysis were performed on trend test under multilinear regression. Results were shown as beta ± se. *P* <0.05 were in bold. OGTT: Oral glucose tolerance test.

Supplemental Table 3. The SNPs in TSK gene region with serum TSK level in obesity subjects.

| SNP | Chromosome | Base position | Minor allele | Minor allele frequency | Beta&95% CI | P |
| --- | --- | --- | --- | --- | --- | --- |
| rs11236956 | 11 | 76538374 | G | 0.41 | 0.17(0.07,0.26) | 0.0007 |
| rs1660579 | 11 | 76537114 | G | 0.43 | 0.17(0.07,0.26) | 0.0007 |
| rs11236955 | 11 | 76538369 | G | 0.41 | 0.17(0.07,0.26) | 0.0007 |
| rs80225615 | 11 | 76538711 | A | 0.33 | -0.15(-0.26, -0.05) | 0.0064 |
| rs11236949 | 11 | 76527066 | C | 0.45 | -0.12(-0.21, -0.03) | 0.0112 |
| rs1149612 | 11 | 76496736 | C | 0.41 | -0.11(-0.21, -0.02) | 0.0196 |
| rs11236946 | 11 | 76519194 | C | 0.04 | -0.25(-0.45, -0.04) | 0.0212 |
| rs1224955 | 11 | 76493945 | A | 0.41 | -0.11(-0.20, -0.02) | 0.0248 |
| rs2451908 | 11 | 76532820 | T | 0.37 | 0.11(0.02,0.20) | 0.0256 |
| rs7128590 | 11 | 76521745 | C | 0.04 | -0.23(-0.42, -0.03) | 0.0273 |
| rs7930972 | 11 | 76527798 | T | 0.08 | -0.22(-0.41, -0.02) | 0.0337 |

rs11236956, rs1660579 and rs11236955 were in high LD value (D’=1, r^2^>0.99). Only SNPs with significant level P < 0.05 were listed. CI: Confidence interval.

Supplemental Table 4. The metabolic traits of subjects carrying different rs11236956 genotypes

|  | **AA (n=3843)** | **GA (n=5246)** | **GG (n=1932)** |
| --- | --- | --- | --- |
| Gender(male%) | 41.84% | 40.75% | 42.65% |
| Age(year) | 56.91±7.10 | 56.87±7.14 | 56.75±6.97 |
| Body mass index(kg/m^2^) | 24.96±3.17 | 25.02±3.30 | 25.12±3.18 |
| Waist circumference(cm) | 84.49±9.20 | 84.53±9.63 | 84.88±9.18 |
| Visceral fat area(cm^2^) | 77.01±36.49 | 81.38±43.23 | 81.89±42.50 |
| Subcutaneous fat area(cm^2^) | 166.31±68.93 | 159.92±63.16 | 165.99±62.50 |
| Fast plasma glucose(mmol/L) | 6.22±1.45 | 6.25±1.52 | 6.29±1.58 |
| 30 minutes plasma glucose after OGTT(mmol/L) | 10.45±2.30 | 10.58±2.40 | 10.63±2.38 |
| 2 hours plasma glucose after OGTT(mmol/L) | 8.85±4.05 | 9.08±4.18 | 9.21±4.21 |
| Fast plasma insulin(mU/L) | 9.50±20.66 | 9.69±19.83 | 9.25±15.49 |
| 30 minutes insulin after OGTT(mmol/L) | 56.66±46.82 | 56.25±45.12 | 56.10±47.12 |
| 2 hours plasma insulin after OGTT(mmol/L) | 55.14±50.46 | 57.48±51.57 | 57.48±50.13 |
| Stumvoll 1^st^ index | 375.45±217.73 | 357.70±211.36 | 351.75±204.80 |
| Stumvoll 2^nd^ index | 128.97±61.08 | 122.62±61.12 | 121.03±59.00 |
| Gutt index | 71.88±17.44 | 71.51±17.57 | 71.05±17.85 |
| Free fatty acid (mmol/L) | 554.79±227.87 | 572.96±239.21 | 575.25±232.51 |
| Total cholesterol(mmol/L) | 5.16±1.00 | 5.15±0.96 | 5.17±0.99 |
| Total triglyceride(mmol/L) | 1.69±1.46 | 1.73±1.59 | 1.77±1.70 |
| Low density lipoprotein-cholesterol(mmol/L) | 3.12±0.80 | 3.11±0.79 | 3.14±0.80 |
| High density lipoprotein-cholesterol(mmol/L) | 1.29±0.35 | 1.30±0.33 | 1.28±0.33 |
| γ-glutamyltransferase(U) | 31.29±32.80 | 32.86±37.4 | 35.98±69.87 |

Data are shown as mean ± standard deviation or N%. OGTT: Oral glucose tolerance test.

Supplemental Figure 1. Flow diagram of patient selection.


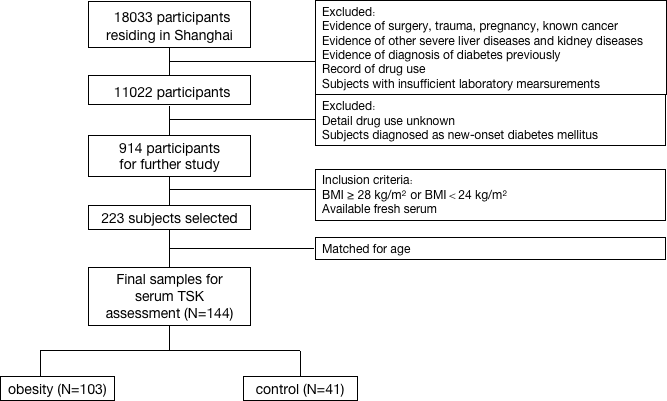


Supplemental Figure 2. Serum TSK levels in human.


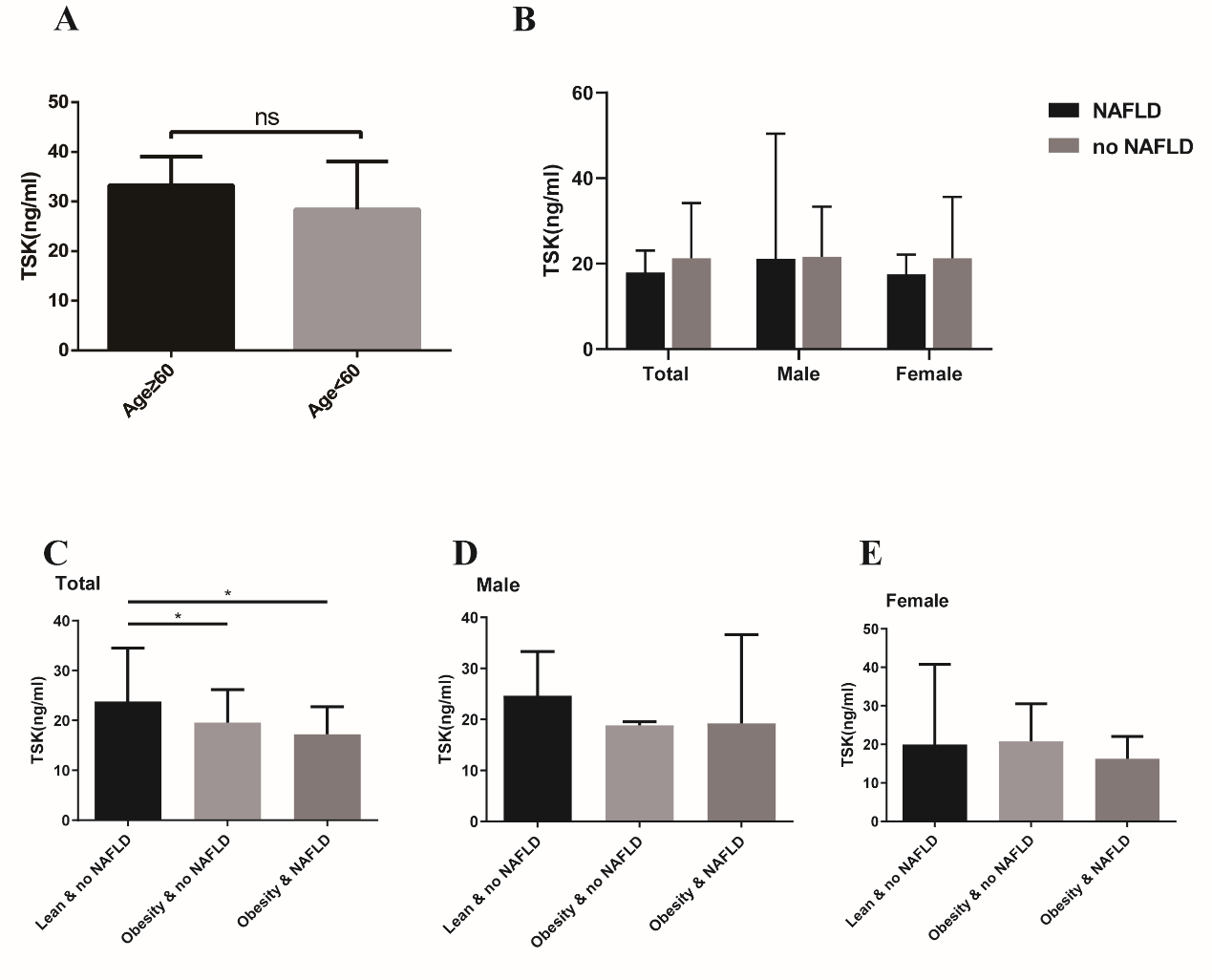


A) Comparison of serum TSK levels between elder group (age≥60, N=105) and others（N=39）, P=0.6604; B) Comparison of the serum TSK level between NAFLD (N=73) and no NAFLD (N=71) group in total population, males (NAFLD: N=10, no NAFLD: N=38) and females (NAFLD: N=63, no NAFLD: N=33), respectively. C-E) Comparison of the serum TSK level among lean and no NAFLD group (N=41 for total,30 for males, 11 for females, respectively), obesity and no NAFLD group (N=30,8,22, respectively), obesity and NAFLD group (N=73,10,63, respectively) in total population, males and females, respectively. Data are shown as median and interquartile range in histogram. Significance of differences was calculated using the non-parametric test. *P<0.05, ** P <0.01, *** P <0.001.

Supplemental Figure 3. The association of loci in *TSKU* gene region with serum TSK level


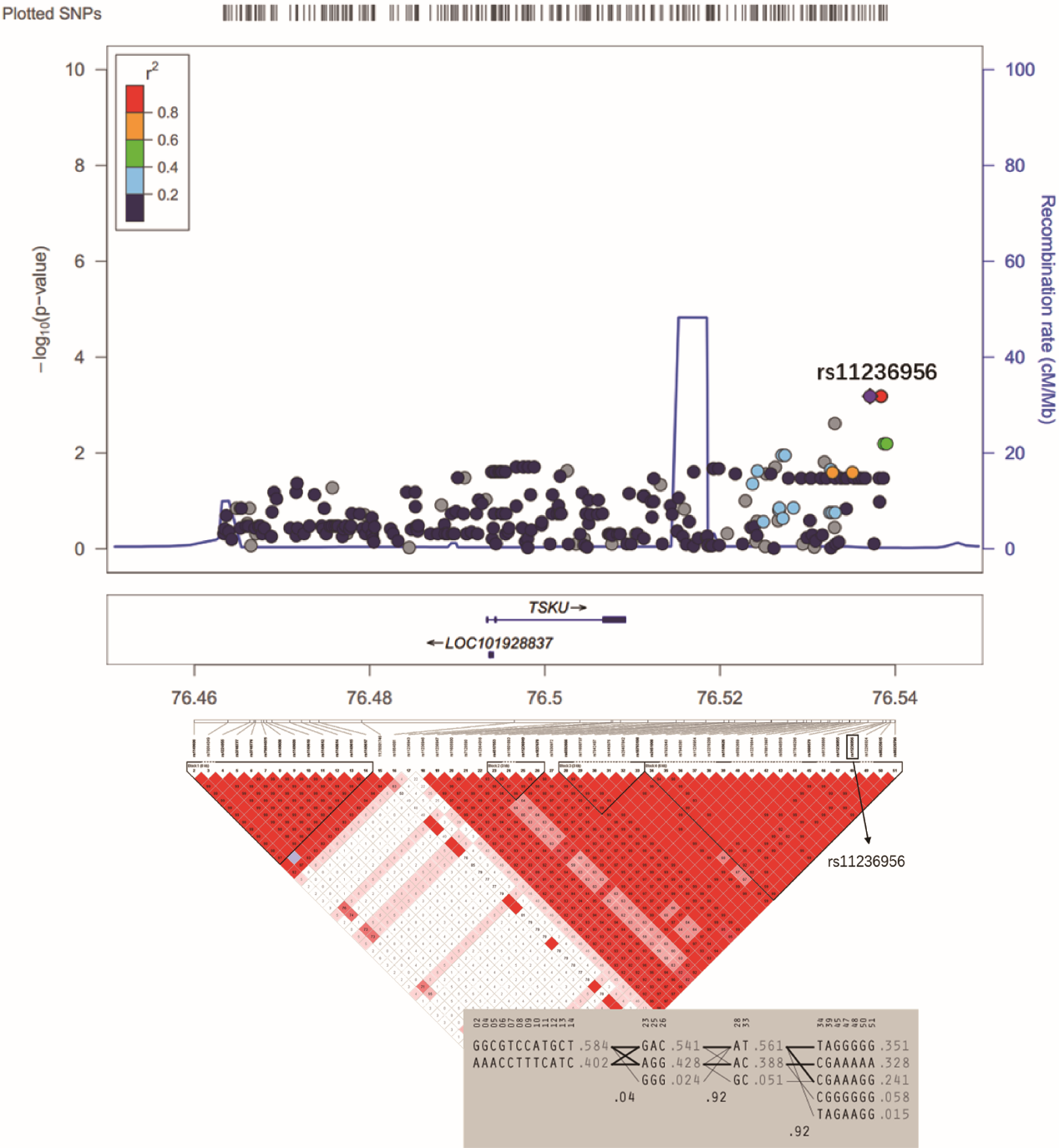


Genome Build is according to hg19, and LD population is based on Asian population in 1000 Genomes. Linkage disequilibrium of variants in TSKU gene region: the region mainly has 4 haplotype blocks.

Supplemental Figure 4. Tissue-specific chromatin states spanning the position of rs11236956

rs11236956
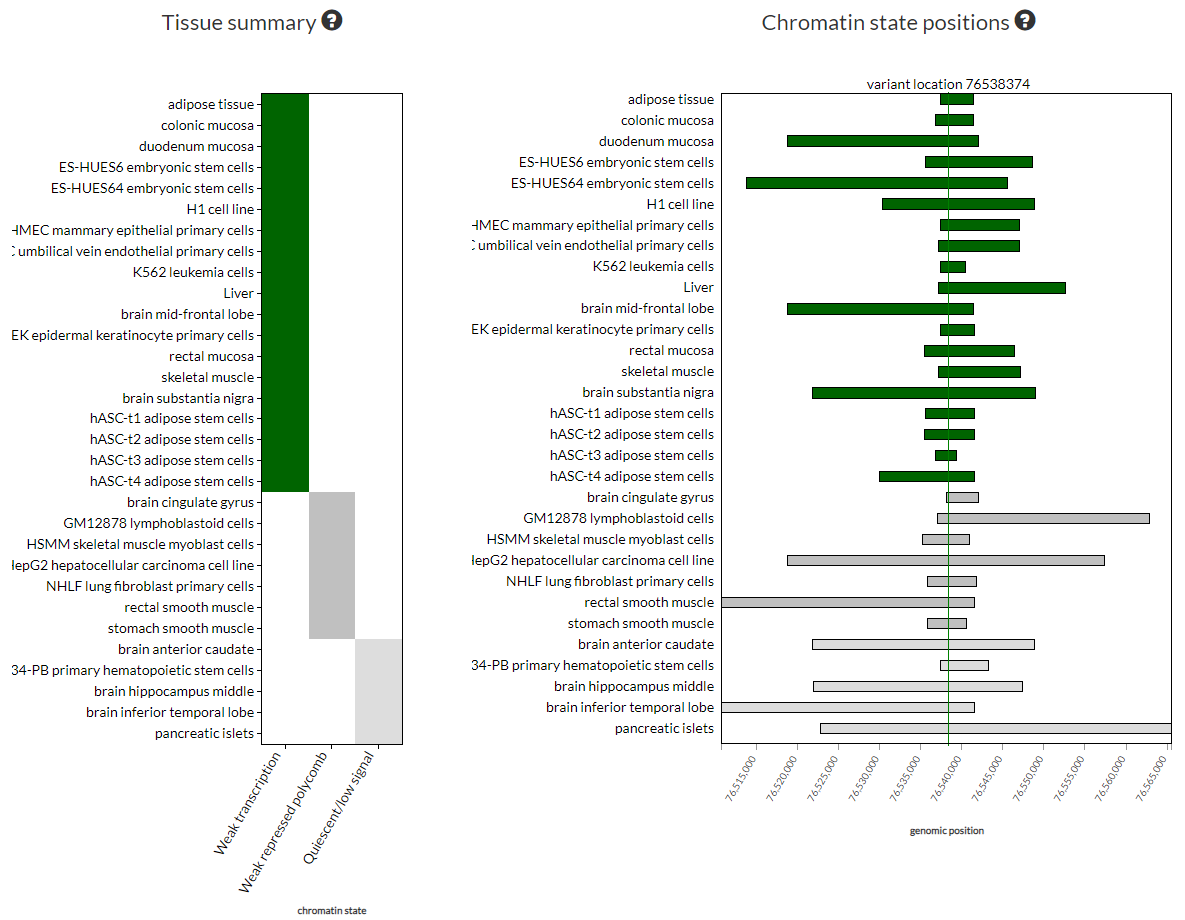


Epigenomic features indicated the potential regulatory role of rs11236956 in a specific tissue or cell type.
